# Multifaceted Shape Memory Polymer Technology for Biomedical Application: Combining Self-Softening and Stretchability Properties

**DOI:** 10.3390/polym15214226

**Published:** 2023-10-25

**Authors:** Chandani Chitrakar, Marc Anthony Torres, Pedro Emanuel Rocha-Flores, Qichan Hu, Melanie Ecker

**Affiliations:** 1 Department of Biomedical Engineering, University of North Texas, Denton, TX 76203, USA; 2 Department of Bioengineering, The University of Texas at Dallas, Richardson, TX 75080, USA

**Keywords:** thiol-ene/acrylate polymer, shape memory polymer, stretchable polymer, flexible polymer, polymer characterization, conformal polymer, self-softening polymer

## Abstract

Thiol-ene polymers are a promising class of biomaterials with a wide range of potential applications, including organs-on-a-chip, microfluidics, drug delivery, and wound healing. These polymers offer flexibility, softening, and shape memory properties. However, they often lack the inherent stretchability required for wearable or implantable devices. This study investigated the incorporation of di-acrylate chain extenders to improve the stretchability and conformability of those flexible thiol-ene polymers. Thiol-ene/acrylate polymers were synthesized using 1,3,5-triallyl-1,3,5-triazine-2,4,6(1H,3H,5H)-trione (TATATO), Trimethylolpropanetris (3-mercaptopropionate) (TMTMP), and Polyethylene Glycol Diacrylate (PEGDA) with different molecular weights (Mn 250 and Mn 575). Fourier Transform Infrared (FTIR) spectroscopy confirmed the complete reaction among the monomers. Uniaxial tensile testing demonstrated the softening and stretching capability of the polymers. The Young’s Modulus dropped from 1.12 GPa to 260 MPa upon adding 5 wt% PEGDA 575, indicating that the polymer softened. The Young’s Modulus was further reduced to 15 MPa under physiologic conditions. The fracture strain, a measure of stretchability, increased from 55% to 92% with the addition of 5 wt% PEGDA 575. A thermomechanical analysis further confirmed that PEGDA could be used to tune the polymer’s glass transition temperature (*T_g_*). Moreover, our polymer exhibited shape memory properties. Our results suggested that thiol-ene/acrylate polymers are a promising new class of materials for biomedical applications requiring flexibility, stretchability, and shape memory properties.

## 1. Introduction

The thiol-ene reaction has attracted researchers in the synthesis area, mainly because of its facile and versatile radical polymerization and click chemistry. Radical polymerization can be mediated by nucleophiles, acids, and bases, resulting in rapid polymerization rates close to 100% monomer conversion [1,2]. The thiol-ene reaction, described in later sections, is a photochemically induced radical step-growth polymerization reaction. Thiol-ene photo crosslinked reactions have been employed in various applications like wound healing, drug delivery, and tissue engineering, as well as in microfluidic applications [2,3,4]. Moreover, the thiol-ene/acrylate polymer system based on TATATO, TMTMTP, and Tricyclodecane dimethanol diacrylate (TCMDA) has displayed a thermally induced shape memory effect, softening property, and biocompatibility [5,6]. Additionally, the improved adhesion of this polymer with noble metals made it a promising candidate for the fabrication of intracortical probes. It has also been studied for potential applications such as vascular stents, self-tightening sutures, and neuroprosthetics [7]. Besides those applications, self-softening variable stiffness Shape Memory Polymers (SMPs) and composites have been extensively researched in the area of soft robotics to be used as microelectronic sensors, actuators, and for shape-morphing adaptive robots [8,9]. Similarly, stretchable SMPs have been used to fabricate a new generation of stretchable electrodes. Yoon et al. demonstrated that transparent and stretchable electrodes can be fabricated by utilizing a pre-strained substrate comprising SMPs and silver nanofibers [10]. Another study by Deng et al. highlighted the significance of stretchable SMPs with a near-body recovery temperature. The team synthesized stretchable electroactive SMPs for promoting proliferation and myogenic differentiation for skeletal muscle tissue engineering [11].

While individual research efforts have investigated SMPs with either self-softening characteristics or stretchability, there is currently a gap in the research, as no study has been undertaken to synthesize SMPs that possess the essential attributes necessary for biomedical applications, including flexibility, stretchability, and self-softening. Hence, this research paper aims to bridge this gap by focusing on synthesizing and characterizing a novel polymer with unique self-softening, flexibility, stretchability, and shape memory properties.

The self-softening property of polymers enables stiff polymers at room temperature to become more pliable when subjected to external stimuli, such as changes in temperature, pressure, or other environmental factors (e.g., moisture and pH). The decrease in the modulus during softening helps to reduce tissue damage and scar formation by addressing the mechanical mismatch between the tissue and polymer substrate. This feature also makes the polymer ideal for bioelectronic devices, as it can conform to organs with irregular surfaces or curvatures, such as the gastrointestinal (GI) tract, maximizing the contact area of the device to the targeted tissue. By facilitating an improved tissue interface, it can reduce the electrode–skin contact impedance and enhance the acquisition and recording of bodily signals [12].

Given that many body organs undergo various degrees of motion, a polymer substrate with stretchability enables it to effectively follow the movement of the organs and adjust to deformations. Combining the self-softening property with stretchability imparts conformability to the polymer. This conformability ensures a close interface of the polymer to the tissue/organ, thereby reducing the motion artifact [13,14,15]. This improvement further results in an enhanced signal-to-noise ratio, especially in the context of bioelectronics during the chronic recording of neural signals.

Furthermore, the shape memory property exhibited by the polymer enables it to restore its initial configuration post-deformation. This mechanism is activated by external stimuli such as heat. This quality makes it ideal for developing minimally invasive devices implanted in a folded state and then recovering their original/expanded shape at the target location [16]. This shape memory polymer can also be potentially used in the field of intestinal anastomosis by offering a sutureless alternative [17]. The controllable shape transformation of this SMP can be utilized to devise a bandage-like device to mitigate anastomotic leakage complications.

To synthesize a polymer possessing all these attributes, including self-softening, stretchability, and shape memory properties, we employed thiol-ene polymer comprising TATATO/TMTMP due to its facile nature of synthesis, shape memory effect, and biocompatibility. To enhance the stretchability of the TATATO/TMTMP polymer, we introduced di-acrylate crosslinkers, i.e., PEGDA, of two different average molecular numbers (Mn 250 and Mn 575) into the thiol-ene polymer. PEGDA, a widely used photocurable polymer, has been employed in the fields of drug delivery, tissue engineering, and biosensing, etc. PEGDA has displayed hydrophilicity and low immunogenic properties. The mechanical properties of the polymer can readily be tuned by adding varying concentrations or different molecular weights of PEGDA. Studies have shown that incorporating shorter-chained PEGDA increases the degree of crosslinking, while longer-chained PEGDA enhances the elasticity of the polymer blend [18]. Furthermore, they have also exhibited the ability to regulate the *T_g_* of the entire material [19]. In this paper, the polymer containing TATATO/TMTMP/PEGDA Mn 250 is referred to as PEGDA 250. Similarly, the one containing TATATO/TMTMP/PEGDA Mn 575 is referred to as PEGDA 575. The TATATO/TMTMP polymer group is used for comparison purposes.

## 2. Materials and Methods

**Figure 1:**
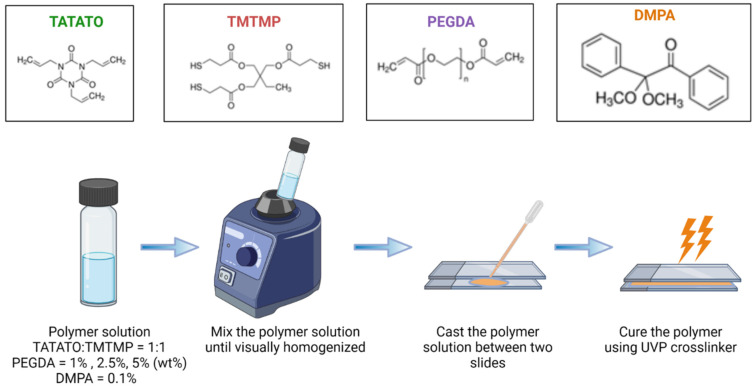
Synthesis process of thiol-ene/acrylate polymer.

1,3,5-triallyl-1,3,5-triazine-2,4,6(1*H*,3*H*,5*H*)-trione (TATATO), Trimethylolpropanetris (3-mercaptopropionate) (TMTMP, Polyethylene glycol Diacrylate (PEGDA) Mn 250 and PEGDA Mn 575, and 2,2-dimethoxy-2-phenyl acetophenone (DMPA) were purchased from Sigma Aldrich (St. Louis, MI, USA). Their chemical structures are presented in Figure 1. The monomers were used to synthesize self-softening, stretchable shape memory polymer without any further purification.

### 2.1. Polymer Synthesis

**Figure 2:**
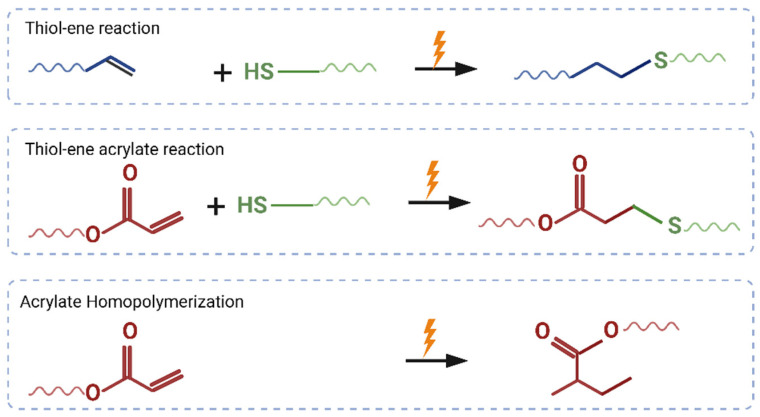
Schematics showing possible ways for thiol-ene acrylate reaction.

A schematic for the polymer synthesis process is shown in Figure 1. The possible mechanisms of reactions during the synthesis process are shown in Figure 2.

**Figure 3:**
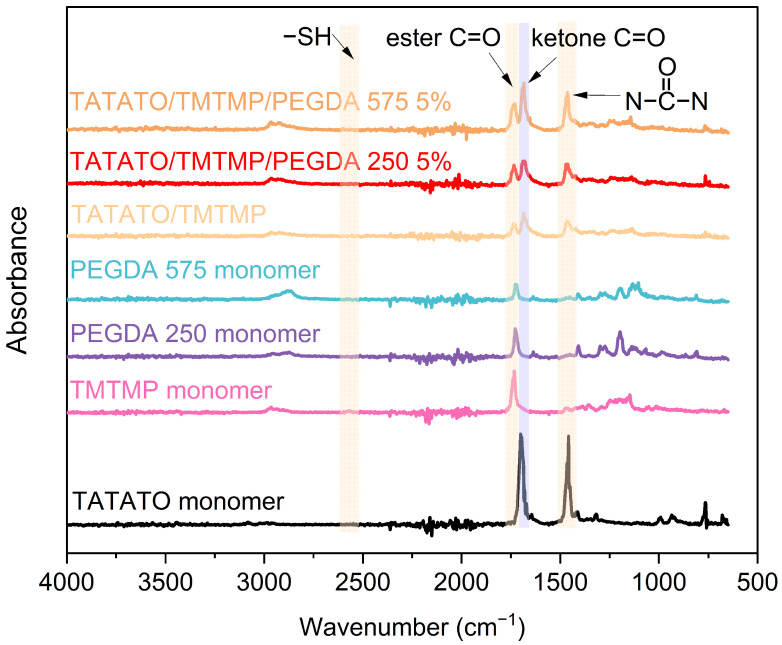
Plot showing FTIR results for monomers and polymer.

Briefly, TATATO and TMTMP were mixed in a molar ratio of 1:1 using 0.1 wt% DMPA as the photoinitiator. Next, PEGDA was added to the solution of TATATO and TMTMP. Different weight percentages of PEGDA Mn 250 and Mn 575 (1%, 2.5%, and 5%) were used to study the behavior of the formulated polymer. The solution was then vortexed using a speed mixer (FlackTek Inc. Model DAC 150.1 FVZ-K) with a speed of 3000 rpm until the solution was visually homogenized. The solution was then cast between two glass slides with spacers (0.3 mm thickness) in between. The polymer was cured for 30 s under the 254 nm band and for an additional 30 min under 365 nm (UVP crosslinker, Analytik Jena, Upland, CA, USA). We utilized different timings at different wavelengths for fully curing the polymer. FTIR was employed to determine the degree of polymer curing. The results obtained from the FTIR (Figure 3) confirmed that the polymer underwent complete curing (determined by the absence of an -SH peak) after 30 s and 30 min of UV exposure at a 254 nm wavelength and 365 nm wavelength, respectively. The cured polymer was then transferred to a vacuum oven (Sheldon Manufacturing Inc., Cornelius, OR, USA, model SVAC2E) set to 120 °C and 20 cm Hg to drive off any unreacted monomer. This post-curing step was performed overnight.

### 2.2. Characterization of Polymer

#### 2.2.1. Fourier Transform Infrared Spectroscopy (FTIR)

Fourier Transform Infrared Spectroscopy (FTIR) was performed on the individual monomers (TATATO, TMTMP, and PEGDA Mn 250 and Mn 575) and the polymer after curing and post-processing. FTIR is a simple and non-destructive method that distinguishes chemical bonds within a material and assesses the interactions between the functional groups in monomers to determine the extent of polymer curing.

Thermo Scientific’s (Waltham, MA, USA) Attenuated Total Reflectance (ATR) FTIR setup was used to collect the spectra from 4000 cm^−1^ to 400 cm^−1^. With the resolution set at 4 cm^−1^, 32 scans were made for all the samples. The FTIR spectra were recorded in the absorbance mode. The background was collected before every set of samples. ATR correction and baseline correction were applied to all the sample spectra afterward.

#### 2.2.2. Swelling

Swelling tests were performed on rectangular 10 mm × 6 mm × 0.5 mm polymer samples with TATATO/TMTMP and PEGDA Mn 575 and Mn 250. The samples were soaked in PBS at 37 °C for 28 days. Initial dry measurements were recorded on day 0 before soaking the samples. The weight increase for the soaked samples was measured at different time points within the month. Before measuring the weight of the soaked polymer, excess water adsorbed onto the polymer was gently removed using KimWipe. The weight gain due to swelling % was determined using the following Equation (1), where *W*_s_ denotes the swollen weight of the polymer while *W_d_* denotes the initial dry weight of the polymer.

(1) $$Weight\;gain\;due\;to\;swelling\left(\%\right)=\frac{{W_{s}-W}_{d}}{W_{d}}*100\%$$

#### 2.2.3. Mechanical Tensile Test

Mechanical tensile testing was performed in dry and soaked conditions using a UniVert tensile tester from CellScale. Two different types of tests were executed, i.e., uniaxial and cyclic mechanical tensile tests. For both measurements, polymer samples were cut out into dog bone shapes with the dimensions provided in ISO 527-2 (5B) [20], as shown in Supplementary Figure S1. The thicknesses of the samples were measured in three different regions within the gauge length. The common thickness value from three different regions was used as the thickness for the calculation. The thickness of the samples fell within the range of (0.2 ± 0.03) mm. The initial distance between the clamps prior to stretching served as the original sample length for the calculations. A 50 N load cell was used for the dry tests, while a 10 N load cell was used for the submersed tests.

Elongation at break, also referred to as fracture strain (*ε_f_*), served as a quantitative measure of the stretchability limit of our polymers. We aimed to understand how well the polymer could accommodate the constant motility of the tissue or organ it would be applied to, whether as a patch, a wearable, or an implant. Therefore, stretchability or elongation at break tests were executed in dry and soaked conditions, mimicking physiologic conditions. Stretchability tests in dry ambient conditions were carried out on polymer samples cut out into dog bone shapes immediately after their removal from the vacuum oven. On the contrary, a submersion stretchability test was performed on dog bone-shaped polymer samples soaked in PBS and placed in a 37 °C incubator for four days before testing to allow for the complete swelling of the polymer. Four days of soaking of the polymer was determined from the swelling test results. The experiment was carried out by setting 0.1 N as the preload and stretching the sample with a 5 mm/min rate until the sample failed or fractured. The Young’s Modulus and failure strain were calculated from the stress–strain curve obtained from the uniaxial elongation tests.

Cyclic tensile testing was also performed to assess the residual deformation and the polymer’s structural durability. The samples in ambient conditions were subjected to stretching in these cyclic tests, reaching 25% and 40% of their total length. The strain ramping was performed at a rate of 16 mm/min, with a 10 s recovery period after each of the five cycles. A preload of 0.1 N was applied at the beginning of every cycle. Cyclic tensile testing was also conducted in a submersion setup to investigate the polymer’s behavior in a soaked state. Similar to the uniaxial elongation experiment in wet conditions, polymer samples were soaked in PBS and placed in a 37 °C incubator for four days. The same parameters as those for the dry cyclic testing, including the preload, ramp rate, number of cycles, and recovery time, were applied in this test. However, only a 25% strain rate was applied to the soaked samples.

For all the tests conducted using the UniVert Tensile Tester, at least five replicates of the test samples were used to assess the reproducibility of the measurements.

#### 2.2.4. Thermomechanical Test

Dynamic Mechanical Analysis is a widely adopted technique for characterizing a polymer’s viscoelastic properties and determining its *T_g_*. This method measures the temperature-dependent properties such as the storage modulus *E*′, loss modulus *E*″, and tan delta, with the latter’s peak indicating the *T_g_*.

A dynamic Mechanical Analyzer (Discovery DMA 850 by TA instruments) was employed to characterize the self-softening property of the polymer. First, the polymer was laser cut into thin rectangular shapes with a length, width, and thickness of 25 mm, 6 mm, and 0.2 mm, respectively, using a Gravograph laser cutter.

Next, both dry and submerged tests were carried out using film clamps and a submersion setup with PBS, respectively. In both test conditions, an oscillation temperature ramp procedure was employed, with a preload of 0.1 N, an oscillation amplitude of 15.0 µm at a 1 Hz frequency, and a temperature ramp from 0 °C to 70 °C. The ramp rate was set at 2.0 °C/min for the dry thermomechanical test. In contrast, the ramp rate was reduced to 1.0 °C/min for the submersion thermomechanical test to allow for temperature equilibrium between the air and the submersion liquid (PBS). Additionally, prior to conducting the submersion test, the samples were soaked overnight at 37 °C.

#### 2.2.5. Shape Memory Characterization

Since thiol-ene/acrylate polymers have been widely known for their shape memory effect [21], qualitative and quantitative shape memory characterization was carried out to examine the shape memory effect of all the polymer formulations. The samples used for these tests were cut into rectangular shapes with a length, width, and thickness of 25 mm, 6 mm, and 0.2 mm, respectively.

A qualitative SMP analysis was performed by initially fixing the polymer into a temporary shape, i.e., a folded configuration. The temporary folded shape was achieved by heating the polymer above the *T_g_* and then placing the folded polymer between two glass slides. The folded polymer was then instantly cooled in a freezer set to −20 °C for around 5 min. Shape recovery % was assessed by letting the polymer recover to its original shape in an aqueous 37 °C environment. A video was captured from the moment the polymer was placed in the 37 °C water until it unfolded to its original shape. MATLAB was used to convert the videos to image frames. An image analysis was conducted using NI Vision Builder software (v2020) to obtain the recovered angle. The shape recovery ratio was calculated using Equation (1), where *θ_r_* is the recovered angle, as shown in Figure S4.

(2) $$Shape\;recovery\;ratio\left(\%\right)=\frac{\theta_{r}}{180{}^{\circ}}$$

The protocol set up for the quantitative SMP analysis test using DMA 850 is summarized in the Table 1 below. Briefly, the polymer was first heated to 50 °C, stretched to a 20% strain of the original length, then cooled to 10 °C. At this state, the shape fixity property was assessed. The polymer sample was heated again to 20 °C and 50 °C to assess the shape recovery effect at room temperature and above the *T_g_*. Shape fixity (*R_f_*) % and shape recovery (*R_r_*) % are calculated for the 1st and 2nd cycle using the following Equations (3) and (4) where *ε_u_*, *ε_m_*, and *ε_p_* denote the strain at unload, maximum strain, and strain at recovery, respectively.

(3) $$R_{f}=\frac{\varepsilon_{u}}{\varepsilon_{m}}\times 100\%$$

(4) $$R_{r}=\frac{\varepsilon_{m}-\varepsilon_{p}}{\varepsilon_{m}}\times 100\%$$

#### 2.2.6. MTT Cytotoxicity Assay

A biocompatibility test is crucial in evaluating materials intended for biomedical device fabrication. One of the biocompatibility tests, the MTT Cytotoxicity Test, is usually conducted to assess a polymer’s toxicity by examining the cell viability when cells are in contact with the material or its extract. In this study, a cytotoxicity test was performed with the cells in direct contact with the polymer, meaning the individual samples were directly placed on the cell layer. Three replicates of each group were employed. Only the polymers containing the highest percentages (5 wt%) of PEGDA Mn 250 and PEGDA Mn 575 were used.

First, all the polymer samples were cut into square shapes measuring 0.45 cm × 0.45 cm and sterilized using 70% ethanol and UV. This 0.20 cm^2^ square polymer adhered to the guidelines of protocol 10993-5, where the sample surface area should be approximately 1/10th of the cell layer surface. Next, approximately 50,000 L929 fibroblast cells were seeded to each well of a 24-well plate and incubated for 24 h at 37 °C. When the cells reached about 70–80% confluency, the square-shaped polymer samples were gently dropped into the center of each well without disturbing the cell layer.

Three wells with only cells and without the polymer samples were used as a positive control. The cells were continued to be cultured for 48 h. Subsequently, 50 µL of a 5 mg/mL MTT solution was added to each well, including the treated wells and control wells. The well plates were returned to incubator for 3 h. After incubation, the medium was carefully removed, and 500 µL of DMSO was added to each well. The plates were foil-wrapped and shaken on a shaker for 10 min to ensure the full dissolution of the formazan crystals formed in active cells. The absorbance was read at OD 570 nm within an hour.

The cell viability was then calculated using Equation (5) provided below, where *OD*_570e_ represents the mean value of the measured optical density of the test sample or experimental group and *OD*_570b_ is the mean value of the measured optical density for the control group. A cell viability exceeding 70% was considered to be indicative of non-cytotoxicity.

(5) $$Cell\;viability\%=\frac{{OD}_{570e}}{{OD}_{570b}}\times 100\%$$

## 3. Results and Discussion

### 3.1. Statistical Analysis

Origin was used to plot the data. A statistical analysis was also performed in Origin. Standard deviation is used for error bars. Analysis of Variance (ANOVA) and the Tukey test were utilized for the statistical analysis. *p* < 0.05 was considered to be statistically significantly different.

### 3.2. FTIR Results

The FTIR results for the monomers and polymer compositions are presented in Figure 3. Only a 5 wt% concentration of polymer with PEGDA 250 and PEGDA 575 was utilized for the test. The figure exhibits the absorption band corresponding to the C=O band (1738 cm^−1^) [22], S-H band (2570 cm^−1^) [23], O=C-N band (1685 cm^−1^) [23], 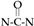 band (1463 cm^−1^) [23], and C=C band (1644 cm^−1^) [24]. The FTIR analysis result also reveals 100% thiol conversion during the polymer synthesis.

### 3.3. Swelling

**Figure 4:**
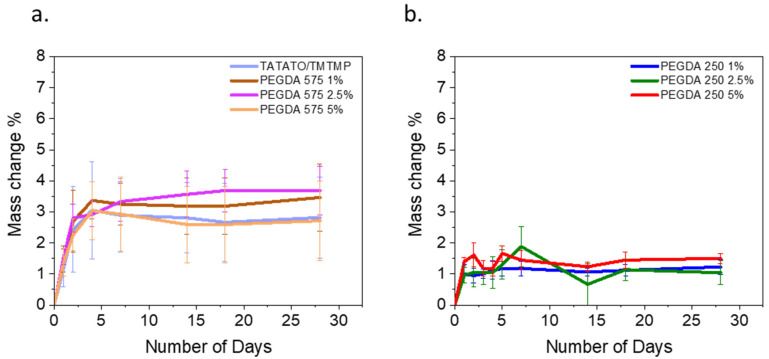
(**a**) Swelling test result over a month for TATATO/TMTMP and PEGDA 575, and (**b**) swelling test result over a month for PEGDA 250.

Figure 4a illustrates the changes in mass over 28 days for all the polymer compositions. In the initial four days, a noticeable mass increase to approximately 3%, attributed to swelling, was observed for all the PEGDA 575 compositions, except for the polymer containing 2.5 wt% PEGDA 575. Following this initial swelling phase, the polymers exhibited stabilization in their mass. However, in the case of the 2.5% PEGDA 575 composition, the mass increase continued to 3.7% until the 7th day, after which, no further changes were observed over the subsequent 28 days.

As anticipated, PEGDA 250, characterized by shorter chains, displayed a lower swelling percentage than PEGDA 575. The highest swelling, nearly 2%, was recorded for the 2.5% PEGDA 250 composition on the 7th day. It is worth noting that there was a similar swelling rate within each group, as the change in PEGDA weight percentage was minimal.

The higher swelling rate observed for PEGDA 575 is attributed to its longer chain length than PEGDA 250, resulting in a lower crosslink density. A reduced crosslink density leads to an increase in the swelling rate [25].

### 3.4. Mechanical Tensile Test

**Figure 5:**
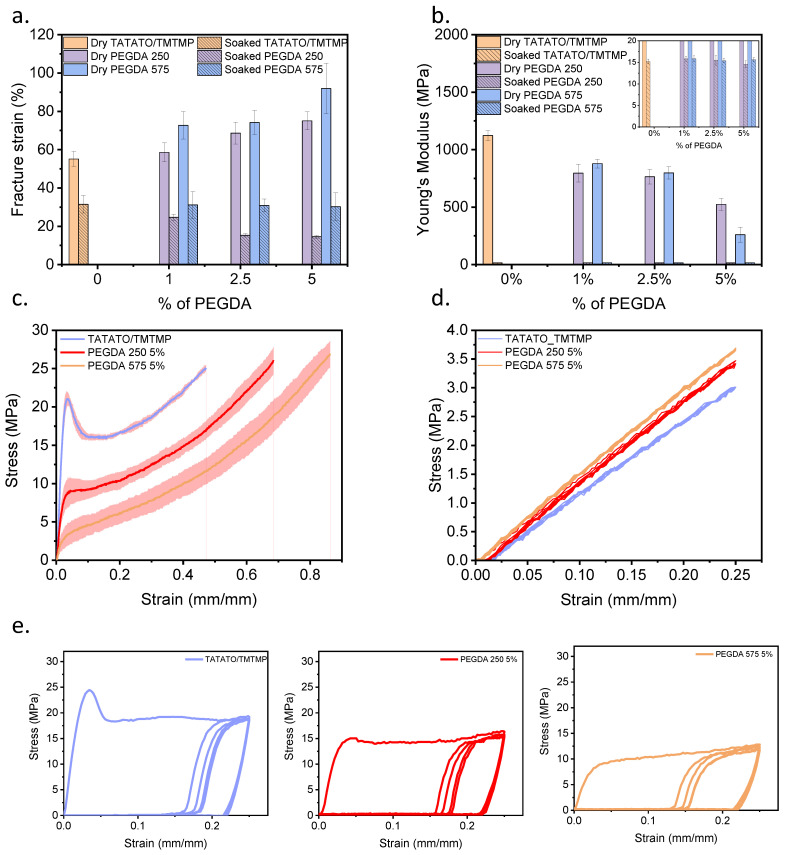
Mechanical tensile testing result of polymers in the dry and soaked state. (**a**) Bar chart comparing the elongation of different compositions of polymers in the dry and soaked states, (**b**) bar chart comparing the Young’s Modulus of the different compositions of polymers in the dry and soaked states, (**c**) strain-stress curves demonstrating the increasing trend in elongation but decrease in Young’s Modulus with the addition of different molecular weight number PEGDA in dry conditions, (**d**) stress–strain curves from the cyclic run of the different compositions of polymer for soaked samples, stretched to 25%, and (**e**) stress–strain curves from the cyclic run for dry polymer samples stretched to 25%. *n* = 5, The data are presented as mean ± standard deviation either as error bars in bar graphics (**a**,**b**) or shaded area around stress-strain curves (**c**,**d**).

Uniaxial mechanical testing for elongation was conducted to assess the polymers’ stretchability; the results are presented in Figure 5a. The stress–strain curves of the polymers with the highest fracture strain within each group are shown in Figure 5c. Adding PEGDA (Mn 250 and Mn 575) improved stretchability compared to the TATATO/TMTMP polymer in its dry state. Notably, the polymer containing PEGDA 575 exhibited a substantial increment in *ε_f_* (*ε_f_* for different wt% of dry PEGDA 575: 72–92%) in comparison to the polymer with PEGDA 250 (*ε_f_* for different wt% of dry PEGDA 250: 58–75%) and TATATO/TMTMP (*ε_f_*: approximately 55%). This improvement in *ε_f_* is attributed to the increased entanglement of polymer chains resulting from the higher molecular weight number of PEGDA. This increased entanglement allows the material to be stretched further before experiencing polymer chain fracture. Furthermore, a clear increasing trend in *ε_f_* (Figure 5a) was evident as the weight percentage of PEGDA (Mn 250 and Mn 575) increased, with PEGDA 575 5% exhibiting a significantly higher *ε_f_* than TATATO/TMTMP.

In contrast, a decline in the stretchability of the polymers in the soaked state was observed compared to that of the dry ones. The *ε_f_* for TATATO/TMTMP was approximately 32%, while for PEGDA 250, it ranged from 14% to 25%, and for PEGDA 575, it was around 31% across all wt% of PEGDA 575. While the presence of water molecules in polymers is expected to enhance their mobility by acting as plasticizers, this phenomenon did not hold true in this context. The reduction in stretchability or fracture strain in the soaked polymers may be attributed to a phenomenon known as “embrittlement induced by swelling of the polymer”, which refers to a significant reduction in the polymer’s ductility due to swelling [26]. Swelling occurs when water molecules diffuse into the bulk polymer. This phenomenon involves the micro-Brownian motion of molecular chains, inducing the local rearrangement of the chains. These rearrangements create both crystalline regions (ordered structures) and non-crystalline/amorphous regions (disordered structures) [19]—consequently, an interface forms between the crystalline and non-crystalline regions, generating a stress field. Additionally, the growth of crystalline regions between the molecular chains impedes chain sliding during stretching, leading to an increased brittleness [27] and a reduction in the *ε_f_* in the soaked state.

Previous studies have also indicated that swelling of a cross-linked polymer network induces pre-strain on the polymer chains [28]. These swelling-induced forces could lead to early rupturing of the polymer chains, consequently reducing the *ε_f_*. Figure 5a additionally illustrates that the soaked polymer with PEGDA 250 showed a declining trend in *ε_f_* as the weight percentage of PEGDA 250 increased. In contrast, the *ε_f_* for the soaked polymer with PEGDA 575 remained constant, irrespective of the weight percentage of PEGDA 575. This could be attributed to the different degrees of homopolymerization for PEGDA 250 vs. 575, but a similar amount of swelling among the various wt%. PEGDA 250 is expected to have a higher degree of homopolymerization due to its shorter chains. Consequently, blocks of acrylate polymer are formed, and the presence of these blocks increases with a higher wt% of PEGDA 250. This results in the polymer becoming more brittle; hence, a declining trend of fracture strain was observed with increasing weight percentages. Conversely, due to the longer chains of PEGDA 575, there was an increased likelihood of network-like structures forming in the polymer with PEGDA 575, resulting in a higher stretchability compared to the PEGDA 250 polymer. Nevertheless, since all the polymer samples with different weight percentages of PEGDA 575 exhibited similar swelling ratios, the stretchability across various weight percentages could have been consistent.

The elastic moduli (Young’s Modulus) of all the samples with different compositions in the dry state and soaked state are shown in Figure 5b. Notably, the dry TATATO/TMTMP polymer exhibited the highest Young’s Modulus of (1.1 ± 0.05) GPa. The introduction of PEGDA (Mn 250 and 575) and an increase in PEGDA concentration (Mn 250 and 575) reduced the Young’s Modulus. The lowest Young’s Modulus (0.26 ± 0.07) GPa was observed for the 5 wt% PEGDA 575. A higher Young’s Modulus is associated with a greater cross-linking density, as it requires more energy to disrupt polymer chains. Including high-molecular-weight PEGDA reduced the cross-linking density and there was an associated decrease in Young’s Modulus [29].

When comparing PEGDA 250 with PEGDA 575, it was anticipated that the Young’s modulus of PEGDA 250 would surpass that of PEGDA 575, attributed to the higher crosslinking density involving PEGDA 250. Though no significant difference (as determined from an ANOVA test with *p* < 0.05 as significant) in Young’s Modulus was observed between PEGDA 250 and PEGDA 575 in the 1% and 2.5% groups, a higher modulus for PEGDA 250 than PEGDA 575 was evident in the 5 wt% group.

No substantial differences in Young’s Modulus were detected upon comparing all the groups of soaked polymers (Figure 5b inset). The Young’s Modulus of all the polymer compositions under physiological conditions remained at approximately 15 MPa, as shown in the Figure 5b inset. This outcome aligns with expectations, as the inclusion of water, acting as a plasticizer, enhances chain mobility, reduces resistance to deformation, and, consequently, leads to a decrease in Young’s Modulus [30,31].

The cyclic tests (dry and soaked) (Figure 5d,e) showed that the polymers could readily endure five cycles. Significant hysteresis in the cyclic testing in the dry state (Figure 5e) was observed following the first stress–strain cycle of testing (loading and unloading) of the dry polymers. This observation indicates that considerable energy dissipated during loading and unloading due to the breakage of bonds [32]. Subsequent cycles, starting from the second, displayed consistent stress–strain behavior with low energy dissipation. Nevertheless, each cycle still exhibited cyclic plastic deformation. Plastic deformation between the first and second cycles, with varying degrees of hysteresis throughout all cycles, demonstrates a distinct loading and unloading path. This deviation arises because the entanglement and disentanglement of polymer chains during cyclic testing do not return to their original positions.

The wet cyclic testing (Figure 5d) also exhibited that the polymers could sustain five cycles. The graph shows that stretching the soaked polymer to a 25% strain did not lead to yielding or plastic deformation and, therefore, was still within the linear elastic regime. No or minimal hysteresis was observed during the cyclic testing in the soaked state, signifying that the soaked polymer followed the same loading and unloading path. This observation may suggest that the soaked/swelled polymer underwent rearrangements in its polymer chain structures, forming crystalline regions.

### 3.5. Thermomechanical Test

**Figure 6:**
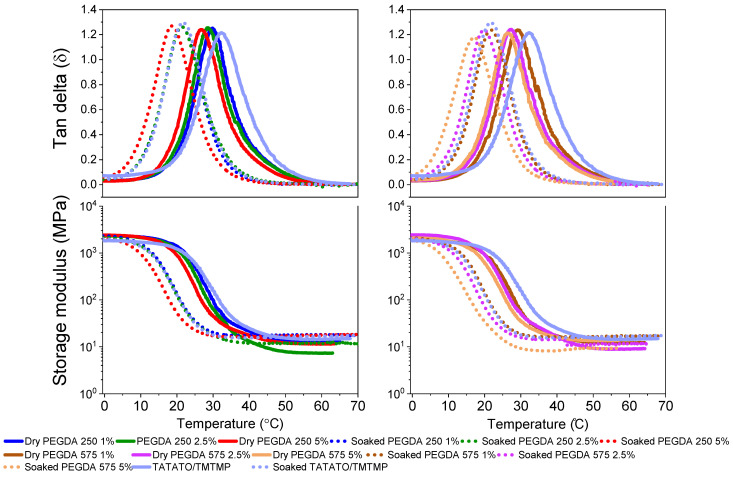
Thermomechanical characterization of thiol-ene/acrylate polymer. The top plots represent the tan delta (*T_g_*), and the bottom plots represent the respective storage moduli of the dry and soaked polymers.

The evaluation of the polymer’s self-softening properties involved a measurement of its glass transition temperature (*T_g_*) in dry and soaked conditions through a Dynamic Mechanical Analysis (DMA). The dry DMA test revealed a decreased *T_g_* by incorporating PEGDA (Figure 6). Introducing long and flexible PEGDA chains into the thiol-ene polymer enhanced the chain mobility, lowering the *T_g_* [33].

Furthermore, measuring the glass transition temperature (*T_g_*) in soaked conditions demonstrated the polymer’s ability to self-soften when exposed to bodily conditions due to water-induced plasticization. This is evident from the shift in *T_g_* toward a lower temperature when measured in the soaked state. For instance, in the DMA results, the *T_g_* of the polymer composition containing 1% PEGDA 575 was found to be 29.5 °C in the dry state (Figure 6). However, in the soaked state, its *T_g_* decreased to 21.9 °C. This change indicates that, at 29.51 °C (the *T_g_* of the dry state), the polymer enters a rubbery state characterized by a lower modulus. This shift in *T_g_* is attributed to the presence of water molecules, which act as plasticizers. When plasticizers are incorporated into the polymer system, they increase the free volume between the polymer chains, allowing the molecular chains to move more freely. Consequently, this reduces the *T_g_* of the polymer in the soaked state [30]. Figure 6 (bottom plots) shows the change in the modulus at different temperatures. The average *T_g_* data for the polymers in both the dry and soaked states are tabulated in Supplementary Tables S1 and S2.

### 3.6. Shape Memory Characterization

**Figure 7:**
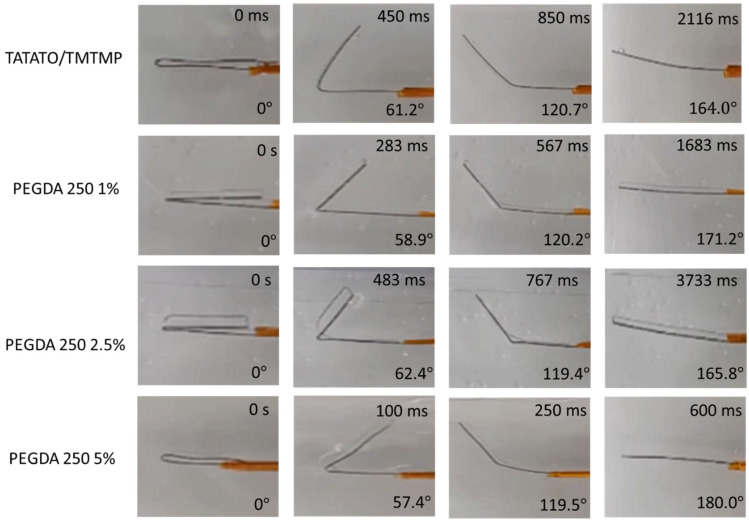

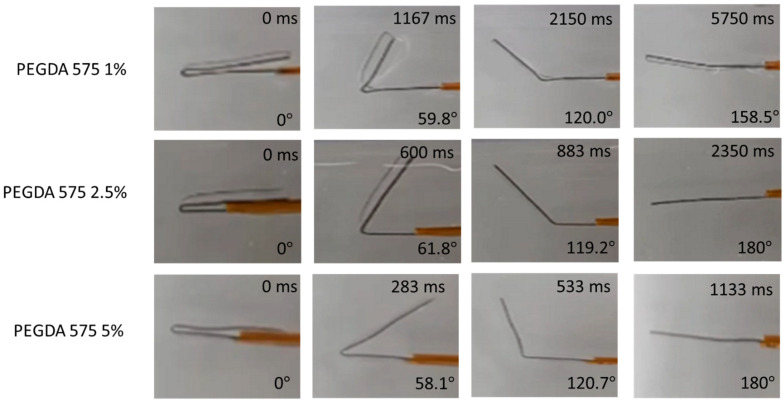
Photographs showing the temporarily fixed shape of polymer (folded configuration) and its subsequent recovery upon placing in an aqueous environment at 37 °C.

A qualitative analysis was conducted to assess the material’s Shape Memory Polymer (SMP) property. The results revealed that all the polymer groups could retain a temporary shape when exposed to temperatures below their *T_g_* but recover to their original shape when subjected to temperatures above *T_g_* (Figure 7). Both Figure 7 and Figure S5 demonstrate the presence of shape memory properties in all the polymer compositions. The qualitative SMP analysis, as shown in Figure S5, also indicated that an increase in PEGDA content led to an enhancement in the shape recovery percentage within each group. This is more pronounced in the PEGDA 575 group. Notably, the PEGDA 575 5% polymer composition exhibited the highest shape recovery percentage of about (100.8 ± 5.5)%. PEGDA 250 5 wt% showed a shape recovery of about (99.7 ± 1.2)%. Though unexpected, we observed the overextending of a few polymer samples, which resulted in a recovery of greater than 100%.

Additionally, the plot reveals that the recovery time for all the polymer compositions was less than a minute. Specifically, PEGDA 250 5% displayed the fastest recovery, taking approximately 1.17 s, while PEGDA 575 1% required about 9.36 s to return to its original shape. From the plot, it can also be observed that the higher shape recovery % polymer composition exhibited a fast recovery. Nevertheless, all the polymer compositions exhibited shape memory properties.

**Figure 8:**
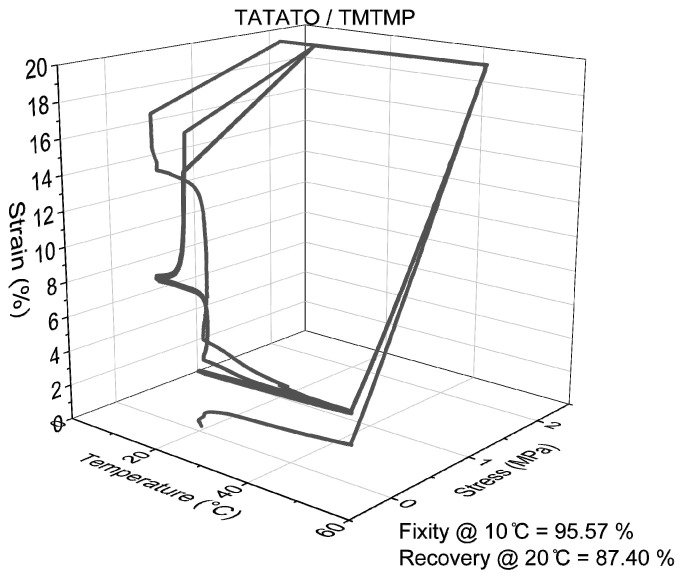

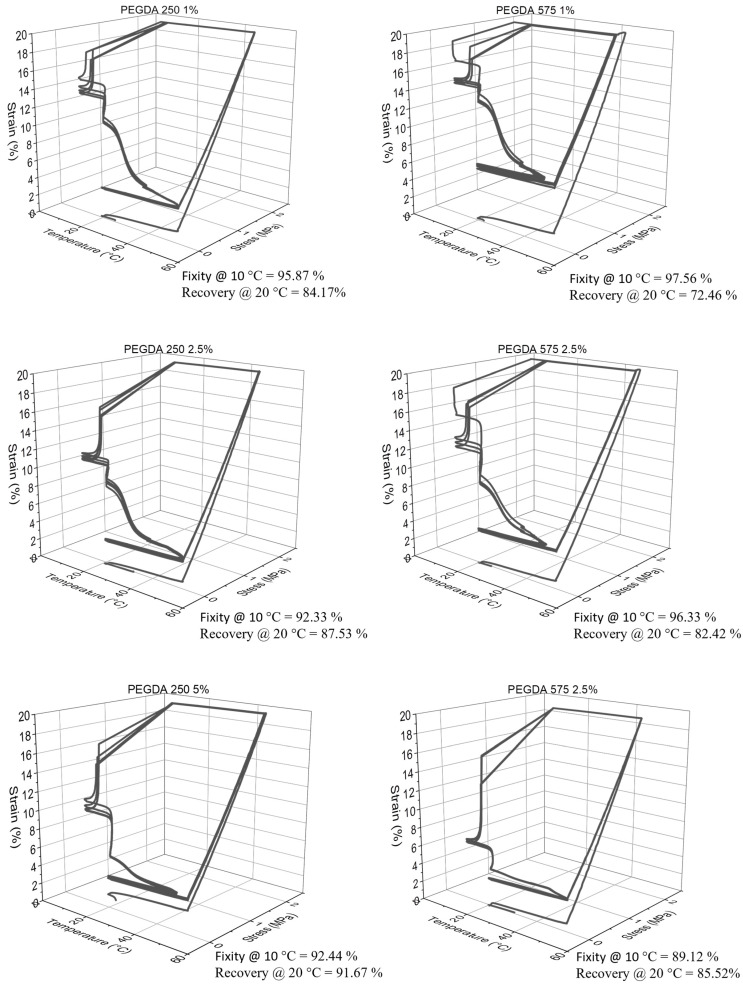
Representative 3D line plot demonstrating shape memory property of all polymer groups.

The quantitative analysis of Shape Memory Polymer (SMP) behavior, conducted using DMA 850, confirmed a consistent pattern in shape recovery, similar to the qualitative analysis. Specifically, an increase in the weight percentage (wt%) of PEGDA correlated with improved shape recovery percentages, as depicted in Figure 8. Conversely, as the wt% of PEGDA increased, a declining trend was observed in shape fixity, also illustrated in Figure 8. Nonetheless, the shape fixity for all the polymer compositions was nearly 90% or higher.

Figure S6 provides additional insights, showing that the shape recovery remained stable and reached nearly 100% after the second cycle (as indicated in Table S3). This underscores the significance of mechanical training in enhancing the shape memory property of polymers [34].

The variation in the shape recovery percentages between the two methods can be attributed to differences in setup, deformation mode (bending vs. stretching), and the distinct mechanisms employed for the shape recovery assessment. As outlined in the methods section, the qualitative shape memory test was conducted within a 37 °C aqueous environment. In contrast, the quantitative shape memory test occurred under dry conditions within a closed, temperature-controlled chamber. In the latter test, the polymer underwent multiple cycles of stress and strain, contributing to the differences observed in the shape recovery percentages.

### 3.7. MTT Cytotoxicity

**Figure 9:**
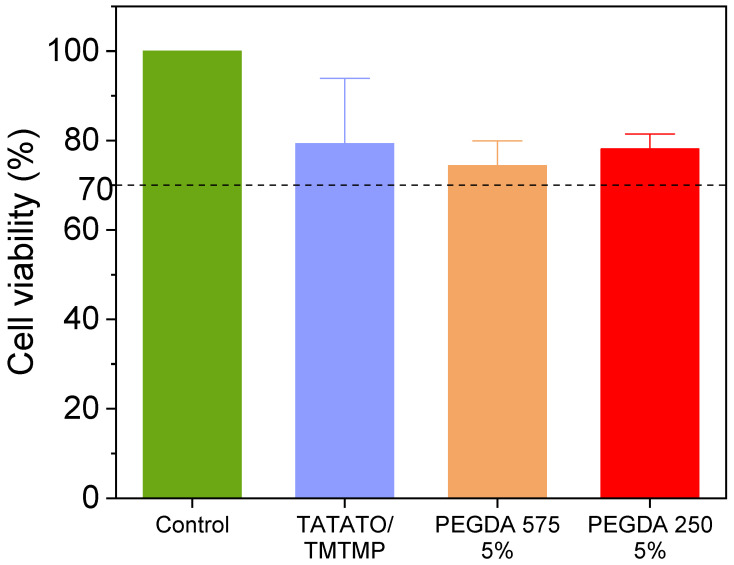
MTT cytotoxicity assay result showing all polymer compositions are biocompatible. Sample size: 3 replicates each group. Dashed line indicates the threshold of 70% above which the material is considered non-cytotoxic.

In Figure 9, the results of the MTT Cytotoxicity assay performed following a 48 h incubation period are presented. The untreated cells (positive control) displayed 100% cell viability. The cell viabilities % of TATATO/TMTMP, PEGDA 575 5%, and PEGDA 250 5% were observed to be (79.3 ± 14.6)%, (74.4 ± 5.5)%, and (78.1 ± 3.35)%, respectively. The reduced cell viability could be caused by leachables (unreacted monomers, photoinitiator) released from the polymer or the pressure from the weight of the samples. Nevertheless, the relative viability is still more than 70% of the positive control group, which exceeded the ISO 10993-5 [35] acceptable threshold of 70%. Consequently, all the groups are categorized as non-cytotoxic.

Although having a negative control is preferable, it is not mandatory, as cytotoxicity is assessed based on a calculation involving the positive control.

## 4. Conclusions

This investigation employed a rapid, solvent-free click chemistry polymerization technique to synthesize thiol-ene/acrylate polymers. Our study showcased the ease of fine-tuning material properties such as Young’s Modulus, stretchability, and glass transition temperature (*T_g_*) by introducing varying amounts of different molecular weights of PEGDA. The initial Young’s Modulus of TATATO/TMTMP stood at (1.1 ± 0.05) GPa, which decreased to (260 ± 65.7) MPa with the addition of PEGDA 575 5 wt%. Furthermore, the Young’s Modulus for all the polymer compositions was reduced to approximately 15 MPa under physiologic conditions. Additionally, we observed a significant enhancement (as determined through ANOVA) in stretchability, ranging from (55.1 ± 3.9)% to about (92 ± 13.1)% without PEGDA and with 5 wt% PEGDA 575, respectively, in dry conditions. These outcomes underscore the polymer’s stretchability and softening capacity upon contact with biological systems. Thermomechanical testing also corroborated the self-softening property by revealing a low storage modulus at 37 °C. Furthermore, the polymer exhibited a remarkable shape memory property, displaying a shape fixity ratio of nearly 90% or greater and a shape recovery ratio exceeding 80% (with an exception for PEGDA 575 1%, which exhibited a shape recovery of 72.5%) at 20 °C. Moreover, the polymers also exhibited low and stable swelling rates over time, and demonstrated non-cytotoxicity to cells.

With the required attributes for biomedical applications such as flexibility, stretchability, and self-softening, this shape memory polymer can be utilized as a self-deployable material that may necessitate minimal invasive surgery if implanted inside the body, as a wound closure device, particularly in anastomosis procedures, as an artificial muscle, as an actuator in soft robotics, or as a substrate for bioelectronic devices—wearable or implantable. Our future studies will be focused on the application of this polymer as a substrate to fabricate flexible, softening, and stretchable bioelectronic devices for neuromodulation. However, there are a few limitations for using this polymer as a substrate for bioelectronic devices. Although this polymer softens greatly under physiological conditions, the transition of the polymer from a high modulus to a low modulus occurs rapidly. This may pose a constraint in the handling time for surgeons during the device’s implantation. This may necessitate the implementation of an insertion guide. Another limitation could involve the need for polymer training to achieve a better recovery % in applications requiring a shape memory effect. While the self-softening and stretchability of this polymer offer advantages, its susceptibility to water absorption may restrict its application as a substrate in implantable devices. Water absorption has the potential to create a leakage path for current to flow, prompting the need for an additional improved encapsulation method to prevent such leakage.

Future polymer-related research endeavors could delve deeper into further exploring the molecular structure of polymers to understand the shape memory mechanism and other properties. While a fast recovery time and stretchability can benefit in the fabrication of high-frequency smart devices in microrobotics, fine-tuning this property to have a greater recovery time can benefit physicians by providing enough time during its handling before it recovers. This could be another avenue worth pursuing. Additionally, the biodegradability property of the polymer also needs to be assessed. Furthermore, upcoming work can involve implementing this polymer to fabricate thin-film bioelectronic devices for biomedical applications, followed by comprehensive in vitro and in vivo characterizations of these devices.

## Figures and Tables

**Table 1:** Shape memory test protocol using DMA 850.

Step	Conditions
Step 0: Conditioning Preload	Apply 0.1 N preload force with force track 125.0% turned on.
Step 1: Isostress	Temperature ramp from 20 °C (~room temperature) to 50 °C (~*Tg* + 20 °C) at a rate of 5 °C/min.
Step 2: Strain ramp	Ramp strain to 20% at a rate of 5.0%/min.
Step 3: Isostrain	Hold the sample at 20% strain for 5 min.
Step 4: Isostrain	With the strain maintained at 20%, ramp down the temperature to 10 °C at a rate of 10 °C/min.
Step 5: Isostress	Hold the sample at 0.1 N for 10 min to assess the shape fixity of the polymer.
Step 6: Isostress	Hold the sample at 0.1 N while ramping the temperature from 10 °C to 20 °C and hold it for 10 min to assess the shape recovery at room temperature.
Step 7: Isostress	Hold the sample at 0.1 N while ramping the temperature from 20 °C to 50 °C and hold it for 5 min to assess the shape recovery at a temperature above *T_g_*.
Step 8: Isostress	Hold the sample at 0.1 N while ramping the temperature from 50 °C to 20 °C to complete one cycle.
Step 9: Conditioning	Repeat all steps three more times.

## Data Availability

The authors confirm that the data supporting the findings of this study are available within the article and its Supplementary Materials.

## Supplementary Materials

The following supporting information can be downloaded at: https://www.mdpi.com/article/10.3390/polym15214226/s1, Figure S1: Specimen dimensions per ISO 572-2 (5B) for tensile testing. All dimensions are in mm.; Figure S2: Cyclic testing of 3 replicates of each polymer composition with PEGDA 250 (Strain to 40%); Figure S3: Cyclic testing of 3 replicates of each polymer composition with PEGDA 575 (Strain to 40%); Figure S4: Shape recovery mechanism of the polymer. θr represents the shape recovery angle for the calculation of shape recovery %; Figure S5: Qualitative SMP analysis. Plot demonstrating the shape recovery % and recovery time for all the polymer compositions; Figure S6: Representative shape memory test data results from DMA 850 for all the polymer groups; Table S1: Comparison of *T*_g_ and storage modulus at different temperatures for different polymer compositions in dry condition; Table S2: Comparison of *T*_g_ and storage modulus at different temperatures for different polymer compositions in soaked condition; Table S3: Calculation of shape fixity and shape recovery for the first and second cycle; Video S1: Qualitative shape recovery for sample with 5% PEGDA 575.
