# Supplementary material for: Multifaceted Shape Memory Polymer Technology for Biomedical Application: Combining Self-Softening and Stretchability Properties
Source: Polymers (Basel). 2023 Oct 25;15(21):4226. doi: 10.3390/polym15214226 (PMC10647621; doi:10.3390/polym15214226)
Supplement: Supplementary file 1 [file polymers-15-04226-s001.zip › polymers-2676145-supplementary.pdf]

Mechanical tensile testing

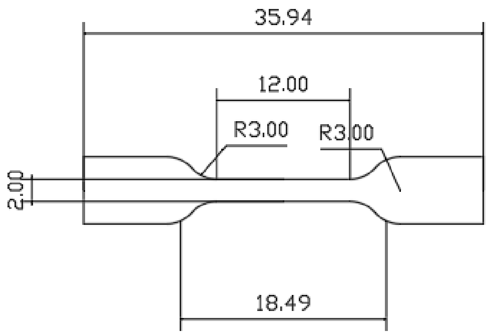

Figure S1. Specimen dimensions per ISO 572-2 (5B) for tensile testing. All dimensions are in mm.

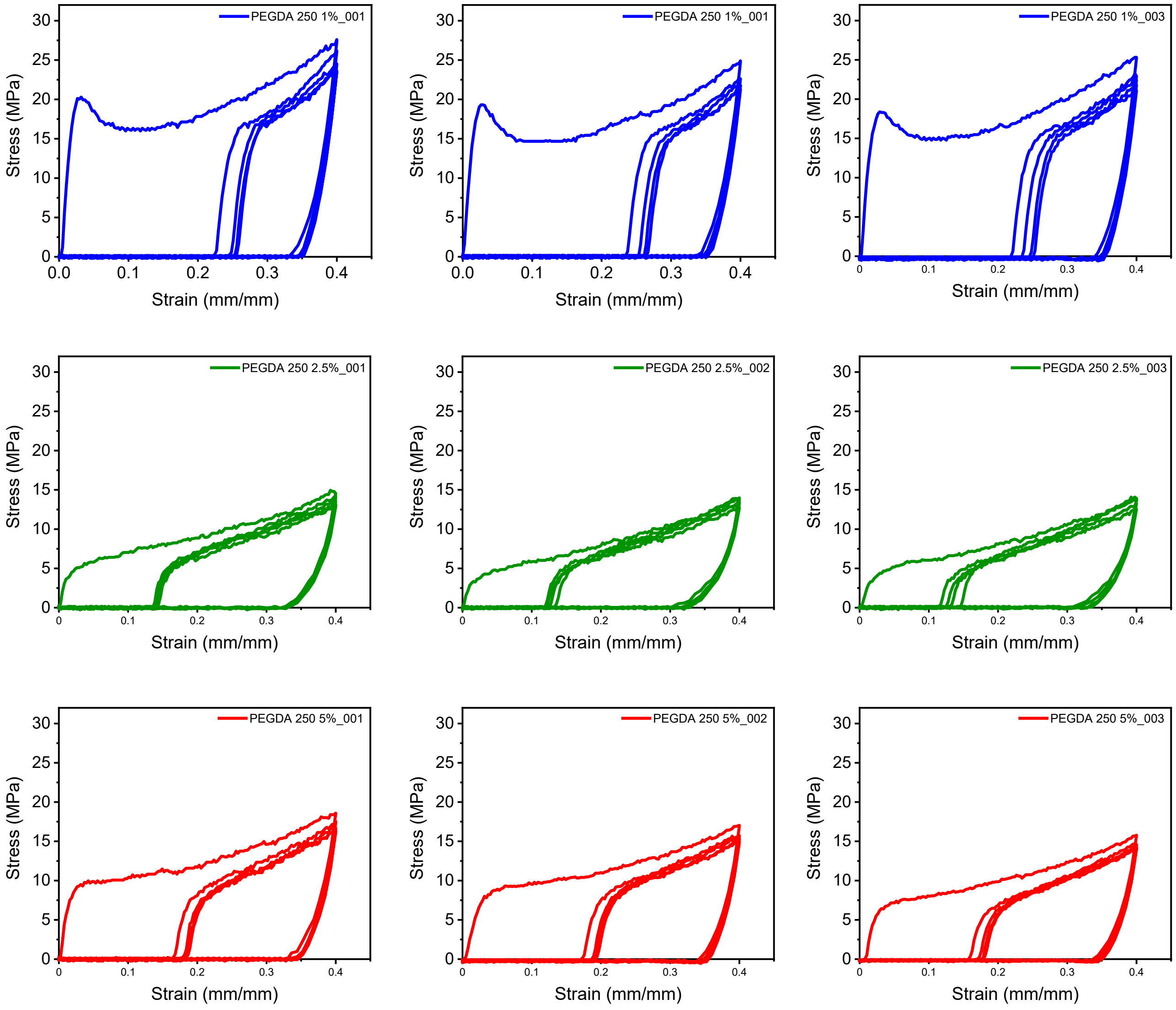

Figure S2. Cyclic testing of 3 replicates of each polymer composition with PEGDA 250 (Strain to 40%).

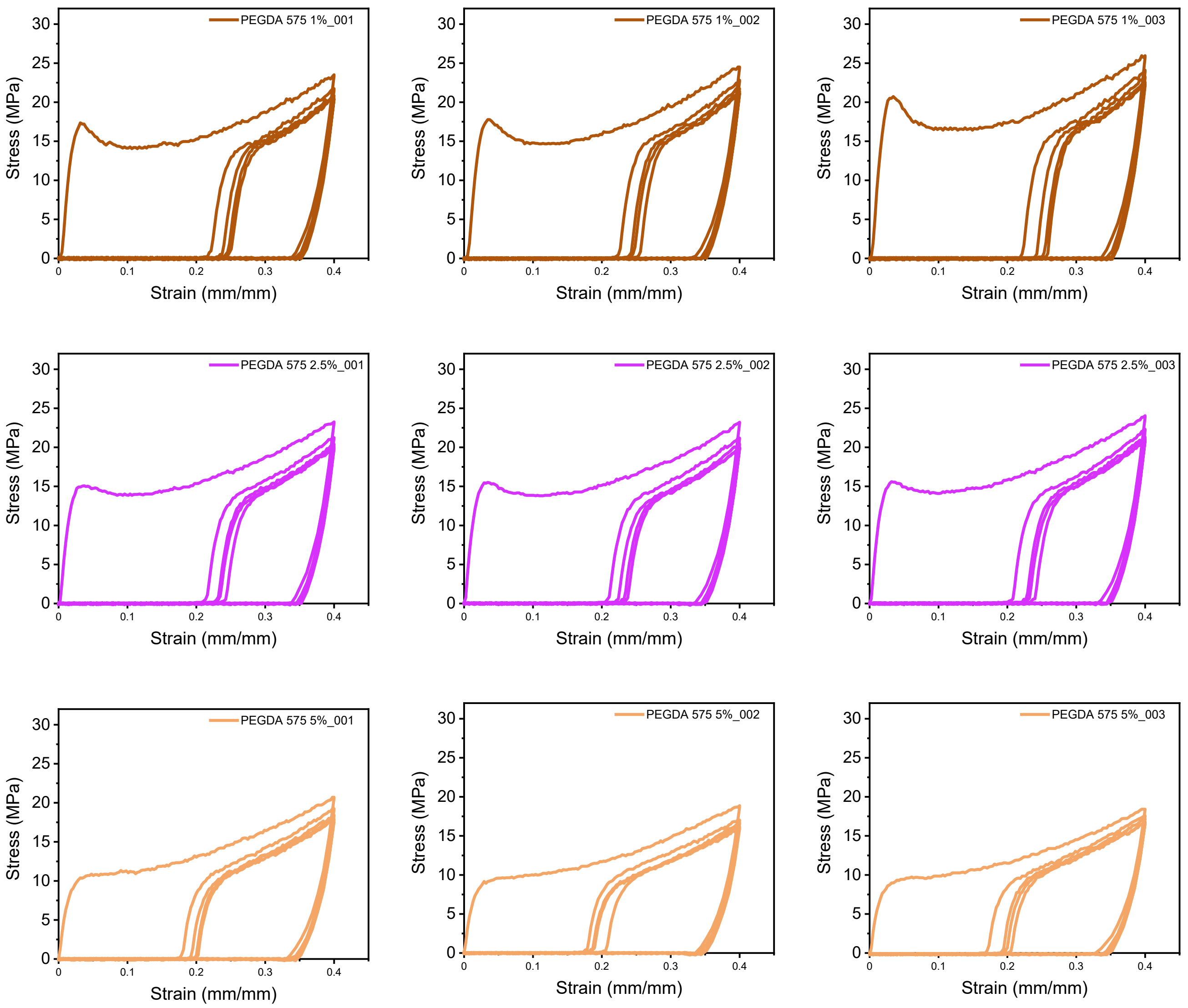

Figure S3. Cyclic testing of 3 replicates of each polymer composition with PEGDA 575 (Strain to 40%).

Thermomechanical Test

Table S1. Comparison of T_g and storage modulus at different temperatures for different polymer compositions in dry condition.

Polymer Composition	T_g (˚C) (Dry)	Glassy modulus at <T_g (10 °C) (MPa)	Storage Modulus at 37 °C (MPa)	Rubbery modulus at > T_g (50 °C) (MPa)
**TATATO/TMTMP**	31.2 ± 1.7	1711.4 ± 277.7	31.0 ± 7.6	16.4 ± 5.1
**PEGDA 250 1%**	29.7 ± 0.4	2282.5 ± 120.5	26.5 ± 2.2	12.5 ± 1.1
**PEGDA 250 2.5%**	28.7 ± 1.0	2114.05 ± 46.7	21.1 ± 3.3	11.76 ± 3.7
**PEGDA 250 5%**	27.0 ± 0.03	2138.3 ± 34.7	20.8 ±0.4	11.7 ± 0.4
**PEGDA 575 1%**	29.2 ± 0.3	1876.04 ±87.9	17.7 ± 3.2	9.2 ± 2.7
**PEGDA 575 2.5%**	28.0 ± 0.6	3425.9 ± 2119	36.1 ± 22.9	19.7 ± 16.6
**PEGDA 575 5%**	26.0 ± 0.6	1839 ± 90.3	18.3 ± 0.6	12.4 ± 1.3

Table S2. Comparison of T_g and storage modulus at different temperatures for different polymer compositions in soaked condition.

Polymer Composition	T_g (˚c) (Soaked)	Glassy Modulus <T_g (10 °C) (MPa)	Storage Modulus at 37 °C (MPa)	Rubbery Modulus >Tg (50 °C) (MPa)
**TATATO/TMTMP**	21.8 ± 0.5	1374.5 ± 44.7	13.1 ± 0.02	13.5 ± 4.4
**PEGDA 250 1%**	21.1 ± 0.8	1384.9 ± 167.0	12.8 ± 6.4	11.6 ± 5.3
**PEGDA 250 2.5%**	21.4 ± 0.8	1370.9 ±100.2	11.9 ±4.4	12.0 ± 5.1
**PEGDA 250 5%**	18.8 ± 0.6	943.6 ±64.4	11.3 ± 4.4	11.7 ± 4.7
**PEGDA 575 1%**	22.0 ± 0.1	1437.7 ± 31.3	9.3 ± 6.2	9.1 ± 6.6
**PEGDA 575 2.5%**	20.4 ± 0.4	1224.3 ±121.4	13.0 ± 3.0	10.3 ± 1.1
**PEGDA 575 5%**	17.4 ± 0.4	708.4 ± 101.1	7.9 ± 5.7	8.2 ± 3.0

Shape Memory Characterization

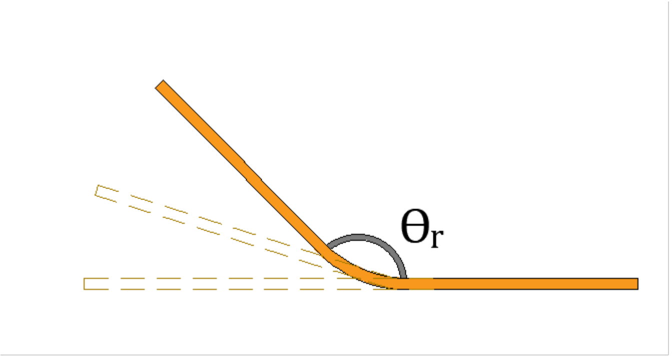

Figure S4. Shape recovery mechanism of the polymer. θ_r represents the shape recovery angle for the calculation of shape recovery %.

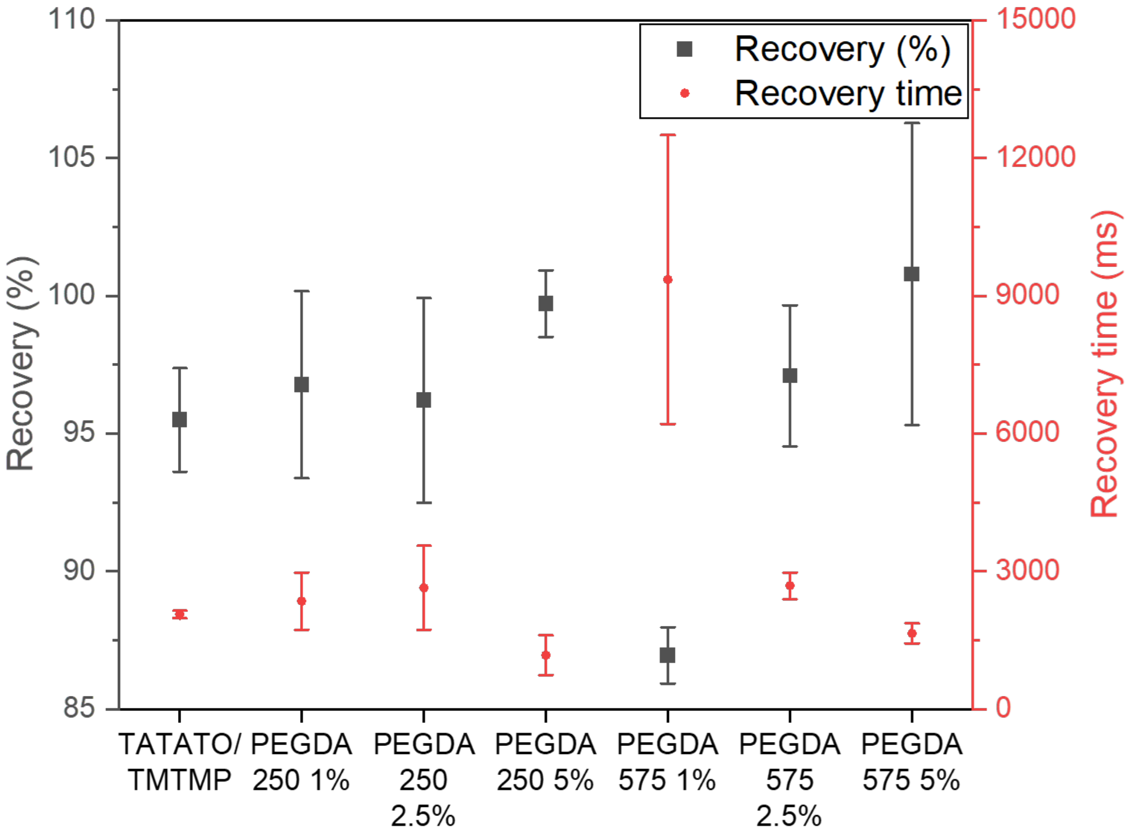

Figure S5. Qualitative SMP analysis. Plot demonstrating the shape recovery % and recovery time for all the polymer compositions. .

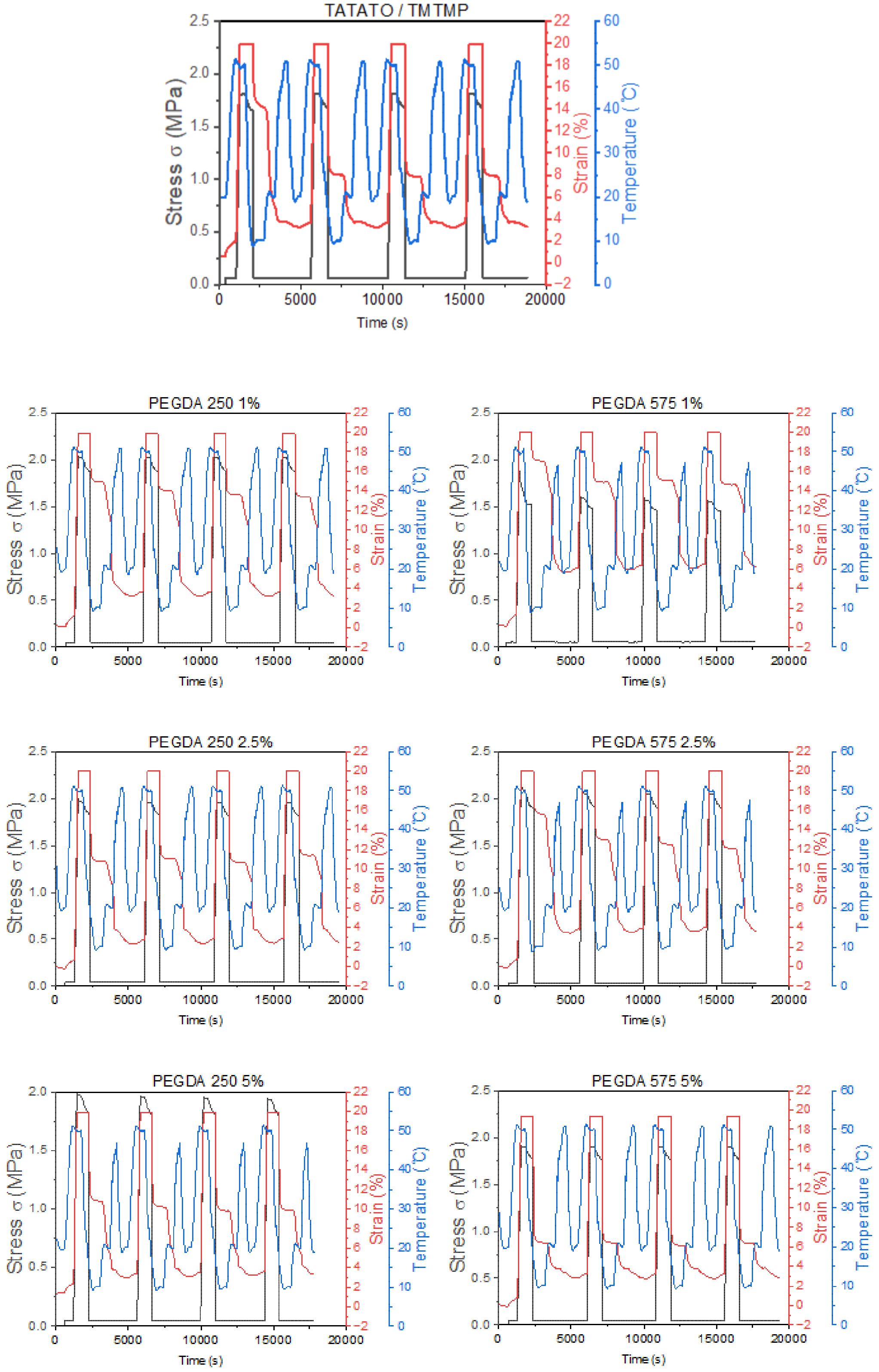

Figure S6. Representative shape memory test data results from DMA 850 for all the polymer groups.

Table S3. Calculation of shape fixity and shape recovery for the first and second cycle.

	TATATO / TMTMP		PEGDA 250 1%		PEGDA 250 2.5%		PEGDA 250 5%		PEGDA 575 1%		PEGDA 575 2.5%		PEGDA 575 5%	
	1st cycle	2nd cycle	1	2	1	2	1	2	1	2	1	2	1	2
L_unload at 10	17.904	16.8783	17.9295	17.7905	17.1762	17.2131	18.2578	18.1545	21.1188	20.7227	19.3958	18.9574	16.448	16.447
L_unload at 20	16.4887	16.2876	17.5121	17.413	16.8377	16.9353	17.4097	17.3938	20.6108	20.4415	18.4389	18.3287	16.0712	16.0638
L_max	18.7332	18.7332	18.7025	18.7025	18.6033	18.6033	19.7504	19.7504	21.646	21.646	20.1341	20.1341	18.4559	18.4559
L recovered at 20	16.1346	16.1313	16.1123	16.1077	15.8611	15.8696	16.9715	16.9886	19.0662	19.1154	17.358	17.3741	15.8932	15.8875
L initial at 20	15.7598	16.1304	15.6252	16.1123	15.4704	15.8611	16.7189	16.9715	18.0857	19.0627	16.7657	17.3522	15.4593	15.8889

Cycle to cycle														
Fixity @10C	95.573634	90.09833	95.86686	95.12365	92.32878	92.52713	92.44268	91.91966	97.56445	95.73455	96.33309	94.15569	89.12055	89.115134
Recovery @ 20C	87.394901	99.96542	84.17119	100.1776	87.52913	99.69003	91.66749	99.38465	72.46019	97.95997	82.41598	99.21277	85.52026	100.05454
